# Obturator nerve entrapment after retropubic tension-free vaginal tape insertion

**DOI:** 10.1007/s00404-022-06704-z

**Published:** 2022-08-22

**Authors:** Christl Reisenauer, Bernhard Kraemer

**Affiliations:** Department of Gynecology and Obstetrics, University Hospital, Tuebingen, Calwerstrasse 7, 72076 Tuebingen, Germany

## Case presentation

After retropubic tension-free vaginal tape (TVT) insertion for stress urinary incontinence, a 52 year old woman reported severe, sharp, right hip and groin pain and thigh muscle weakness, worse on adduction. Prolonged standing or walking was not possible. Electromyography (EMG) revealed an impairment of the right obturator nerve function. The sonographic and radiological examinations were unrevealing, a hematoma was ruled out. Analgesic therapy was ineffective.

**Fig. 1:**
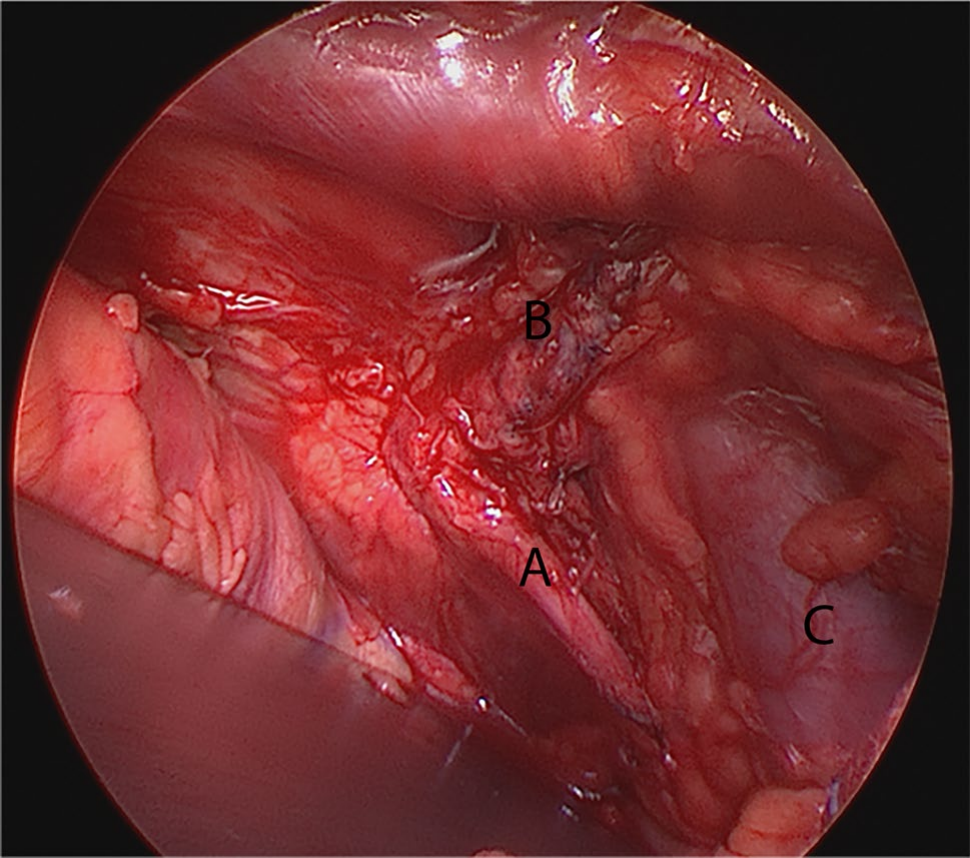
Intraoperative view of the right TVT arm passage through the retropubic space. **A** Obturator nerve, **B** Retropubic TVT arm, **C** Internal iliac vein

The right TVT-arm was partially removed by laparotomy 14 days after the initial surgery and 2 days after referral to our hospital. The woman recovered without motor deficits and maintenance of continence. The obturator nerve entrapment was caused by a too lateral needle passage on the right side. The TVT arm passed the retropubic space lateral to the obturator nerve and medial to the external iliac vein (Fig. 1).

## Discussion

Brubaker et al. showed that neurologic adverse events were more common after transobturator than after retropubic midurethral slings (9.7 versus 5.4%). In their study most neurologic symptoms were mild in nature and had resolved 6 weeks postoperatively [1]. The obturator nerve entrapment is an extremely rare complication and requires surgical intervention.
